# The prevalence and genetic diversity of group A rotaviruses on pig farms in the Mekong Delta region of Vietnam

**DOI:** 10.1016/j.vetmic.2014.02.030

**Published:** 2014-06-04

**Authors:** Pham Hong Anh, Juan J. Carrique-Mas, Nguyen Van Cuong, Ngo Thi Hoa, Nguyet Lam Anh, Do Tien Duy, Vo Be Hien, Phan Vu Tra My, Maia A. Rabaa, Jeremy Farrar, Stephen Baker, Juliet E. Bryant

**Affiliations:** a Oxford University Clinical Research Unit, Hospital for Tropical Diseases, Viet Nam; b Nong Lam University, Thu Duc, Viet Nam; c Sub-Department of Animal Health, Dong Thap, Viet Nam; d Centre for Tropical Medicine, Nuffield Department of Medicine, Oxford University, London, United Kingdom; e University of Edinburgh, London, United Kingdom; f The London School of Hygiene and Tropical Medicine, London, United Kingdom

**Keywords:** Rotaviruses, Pigs, Vietnam

## Abstract

Group A rotaviruses (ARoVs) are a common cause of severe diarrhea among children worldwide and the cause of approximately 45% of pediatric hospitalizations for acute diarrhea in Vietnam. ARoVs are known to cause significant economic losses to livestock producers by reducing growth performance and production efficiencies, however little is known about the implications of asymptomatic endemic circulation of ARoV. We aimed to determine the prevalence and predominant circulating genotypes of ARoVs on pig farms in a southern province of Vietnam. We found overall animal-level and farm-level prevalence of 32.7% (239/730) and 74% (77/104), respectively, and identified six different G types and 4 P types in various combinations (G2, G3, G4, G5, G9, G11 and P[6], P[13], P[23], and P[34]). There was no significant association between ARoV infection and clinical disease in pigs, suggesting that endemic asymptomatic circulation of ARoV may complicate rotavirus disease attribution during outbreaks of diarrhea in swine. Sequence analysis of the detected ARoVs suggested homology to recent human clinical cases and extensive genetic diversity. The epidemiological relevance of these findings for veterinary practitioners and to ongoing pediatric ARoV vaccine initiatives in Vietnam merits further study.

## 1 Introduction

Rotaviruses (RoV) are major pathogens causing severe diarrhea in young mammals and birds of many species. In pigs, RoV are considered an important pathogen due to their significant economic impact and the potential of zoonotic transmission to humans (Midgley et al., 2012). RoV are members of the family Reoviridae, and are non-enveloped viruses with a segmented, double-stranded RNA genome. RoV are classified into eight serogroups (or species) A–H, based on antigenicity of the VP6 protein; groups A–C can infect both humans and animal species (primarily mammals), while groups D–H infect primarily avian species, but are not associated with disease in humans (Dhama et al., 2009; Matthijnssens et al., 2012). From a human medical perspective, group A rotavirus (ARoV) are the most important species of the genus, accounting for >90% of human infections and exhibiting the most evidence for frequent host-switching and traffic between mammalian hosts (Estes and Kapikian, 2007). From a veterinary perspective, however, rotavirus B and C (BRoV and CRoV) are likely to be equally important, particularly in swine where both have been associated with severe diarrhea (Smitalova et al., 2009). Although there are an increasing number of full RoV genomes available for analysis, the configuration of the two primary surface antigens on the outer viral capsid, G(VP7) and P(VP4), still forms the basis of the most widely applied binary classification system for RoV (Matthijnssens et al., 2011). Diversity of VP4 and VP7 proteins are key determinants of immune protection and are highly relevant to vaccine development. G-P classification has been most extensively applied to the ARoVs, where the various G and P combinations tend to be associated with specific host species (Estes and Kapikian, 2007). Among the ARoVs, 27 G genotypes and 34 P genotypes are currently recognized, however, the number continues to expand as more as emphasis is placed on RoV surveillance in non-human species.

Surveillance of circulating RoVs has revealed the presence of uncommon genotypes in humans that are commonly found in domestic animals (Chitambar et al., 2009; Nguyen et al., 2007; Duan et al., 2007; Matsushima et al., 2012), and the presence of viruses with hybrid genome constellations (Park et al., 2011; Wang et al., 2010), suggesting that some ARoVs are able to cross species barriers and contribute to human rotavirus diversity. As part of a larger platform to study zoonotic disease transmission in Vietnam, we surveyed ARoVs in pigs of smallholder farms in the Mekong Delta. We aimed to determine the pig-level and farm-level prevalence of ARoV; to investigate associations between porcine ARoV prevalence, enteric disease in pigs, and risk factors for infection; and to characterize the diversity of porcine ARoVs based on G and P genotypes.

## 2 Materials and methods

### 2.1 Study location and design

The survey was carried out between February and May 2012 in Dong Thap province in southern Vietnam as previously described (Carrique-Mas et al., 2013). The study included 4 of 12 districts (Cao Lanh, Chau Thanh, Hong Ngu and Thanh Binh) from which a census of all registered farms was available. Farm size strata were defined as small (<10 pigs); medium (from 10 to 50 pigs); large (>50 pigs), with approximately 10 farms per stratum, aiming at 120 farms. From each farm, freshly voided individual fecal samples (∼5 g) were randomly collected from 10 pigs. Samples were recorded as diarrheic or not based on visual inspection of fecal consistency. Farmer survey questionnaires were used to collect information on animal and farm characteristics as well as farming practices. The study was approved and implemented by the Sub-Department of Animal Health Dong Thap province and Nong Lam University.

### 2.2 Molecular processing

Fecal RNA was extracted from 200 μL of 10% (w/v) fecal suspensions using MagNA Pure 96 Viral NA small volume kit (Roche) and an automated extractor (Roche). Presence of PCR inhibitors and RNA quality control was assessed by spiking samples with an RNA internal extraction control (Equine Arterivirus) prior to extraction (Scheltinga et al., 2005). The total RNA recovered (60 μL in nuclease free water) was stored at −80 °C until use. cDNA was screened for rotavirus A by realtime RTPCR (gene target NSP3, positions 988–1074, designed to detect all ARoVs) (Freeman et al., 2008) that has previously been validated and used in etiological studies in Vietnam (Dung et al., 2013). Rotavirus outer capsid genes (VP7 and VP4) amplification was performed by conventional RT-PCR using two primer sets per gene as previously described (Tra My et al., 2011). Subsequent to the detection of a novel G26 genotype in several human clinical cases of severe diarrhea from the same study province (manuscript in preparation), an additional set of primers were designed to detect G26 (Forward: ATGTATGGTATTGAATATACCAC; Reverse: GACATRTCTAACTGCAAATCTGA), and were used on all ARoV positive samples. PCR amplicons were visualized on 2% agarose gels under ultraviolet (UV) light after staining with 3% ethidium bromide. All amplicons of the expected bandsize and sufficient intensity were purified and transferred to Macrogene (Seoul, Korea) for commercial capillary sequencing. Forward and reverse reads were generated for each amplicon, and raw sequence output edited within Vector NTI software. The resulting VP4 and VP7 sequences were analyzed using the online automated classification tool RotaC v2.0 (http://www.regatools.be/rota20) for initial genotype determination, and by additional phylogenetic analyses for selected genotypes.

### 2.3 Risk factor analysis

Survey data were analyzed and potential risk factors for rotavirus infection investigated using two-level random effects logistic regression models (univariable and multivariable) as previously described (Carrique-Mas et al., 2013). All statistical analyses were performed using R (http://www.r-project.org/). Random-effects logistic regression modeling was carried out using the lme4 package.

## 3 Results

### 3.1 Farms and number of samples

A total of 104 farms distributed across 4 districts of Dong Thap province were sampled. The distribution of farms sampled by district, and the underlying study population are shown in Table S1. In two of the surveyed districts, very few farms met the criteria for large size (>50 pigs), and thus all available large farms were sampled. The density of pig farms varied considerably across the study districts, with an average of 1.3 farms/km^2^ (greatest in Chau Thanh district with 3.6 farms/km^2^) and lowest in Thanh Binh district (0.5 farms/km^2^).

A total of 1151 individual fecal samples were collected, of which 76 corresponded to pigs with diarrhea. Analysis of samples was conducted on all 76 diarrheic samples, plus 5–6 normal fecal samples randomly selected per farm, for a total of 730 samples (263 from small farms; 270 from medium-sized farms; 197 from large farms).

### 3.2 Prevalence and risk factors for ARoV infection in swine herds

**Fig. 1:**
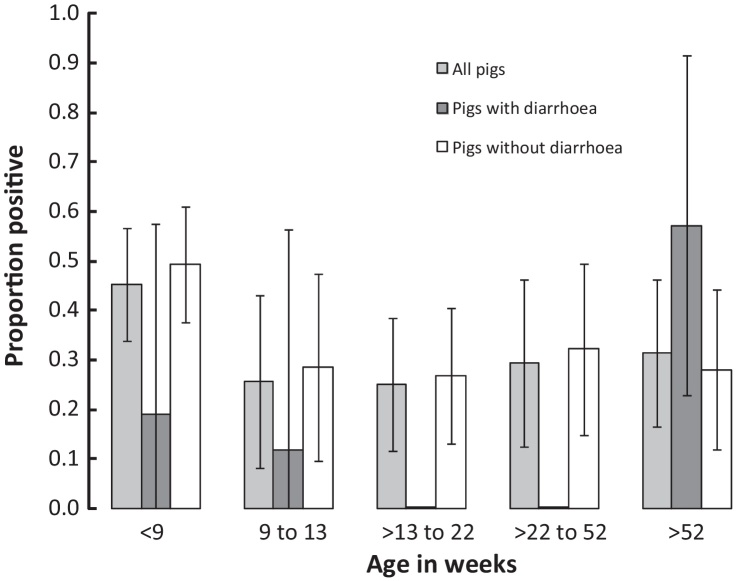
ARoV prevalence by age group (in weeks) and health status.

Overall animal-level and farm-level prevalence of ARoV was 32.7% (239/730) and 74% (77/104), respectively. The prevalence of ARoV infection was 24.9% (163/654) for apparently healthy pigs, and 19.7% (15/76) among diarrheic pigs. The majority of positive samples had low levels of fecal shedding (as estimated by quantitative realtime PCR – *C*_*t*_ > 35). However, a small number of animals had extremely high viral loads, and there was a statistically significant difference between the RoV viral loads in diarrheal pigs (median *C*_*t*_ = 30.6, IQR [25.3–34.6]) and non diarrheal pigs (median *C*_*t*_ = 35.0, IQR [30.8–37.1)] (*p* = 0.002; Kruskal–Wallis test). Notably, the pig with highest ARoV load (*C*_*t*_ = 9) was asymptomatic. The relationship of ARoV positivity and age group (recoded by week in quintiles) is shown in Fig. 1. We found no evidence for geographical clustering of ARoV positive farms.

### 3.3 Risk factors for ARoV infection in pigs

We performed a multivariable logistic regression analyses (both univariable and multivariable models) to investigate and identify risk factors for RoV infection on pig farms, the resulting data are shown in Table 1. Pig age was investigated both as a (log-transformed) continuous and as a categorical binary variable (after recoding the data as quintiles). This variable was finally fitted as binary (<60 days), since it had the best fit. A farm location in Chau Thanh district was the single most significant risk factor (OR = 2.9), followed by ‘pigs aged less than 60 days’ (OR = 2.87), the use of river water as drinking source for pigs (OR = 3.26), ‘farm located in communes of high poultry density’ (OR = 2.45), and the total number of pigs on the farm (OR = 1.44). Census data from the province indicates that Chau Thanh district had the highest density of pigs per km^2^ relative to the other survey districts (Table S1).

### 3.4 G and P genotyping

All ARoV positive samples with *C*_*t*_ < 36 (representing 163 of 255 positive amplicons) were genotyped by partial sequencing and phylogenetic analysis of the VP4 and VP7 genes. A total of 24 and 28 novel G and P sequences were generated, respectively, enabling either full or partial genotyping for 32 of 163 (19.6%) samples (Table 2). Six different G types and 4 P types were identified in various combinations: G3 P[23] (*n* = 1); G4 P[6] (*n* = 9); G11P[13] (*n* = 3); G5 P[13] (*n* = 3); G2 P[34] (*n* = 1); G4 P[unknown] (*n* = 2); G9 P[unknown] (*n* = 2); G unknown P[23] (*n* = 3). One sample tested positive using a pair of G26-specific primers, from which a 425 bp fragment was generated with 93% homology to a porcine G26 strain (Miyazaki et al., 2011). Repeated attempts to extend either the G11-like or the G26-like sequences were not successful, hence these were not included in the subsequent phylogenetic analysis. For the remaining 136 ARoV samples on which typing was attempted, samples either did not yield amplicons or consensus sequencing of amplicons did not yield sufficient quality sequence. More specifically, for VP4 amplifications, 412 PCR reactions yielded 39 amplicons, of which 23 generated usable sequence data (5.5%). For VP7, 411 amplifications yielded 43 amplicons, and only 20 quality sequence reads. Due to the limited typing data, no single G type was dominant across the farm sites. Both P[6] and P[13] were detected across all four surveyed districts.

### 3.5 Sequence and phylogenetic analysis of VP7

**Fig. 2:**
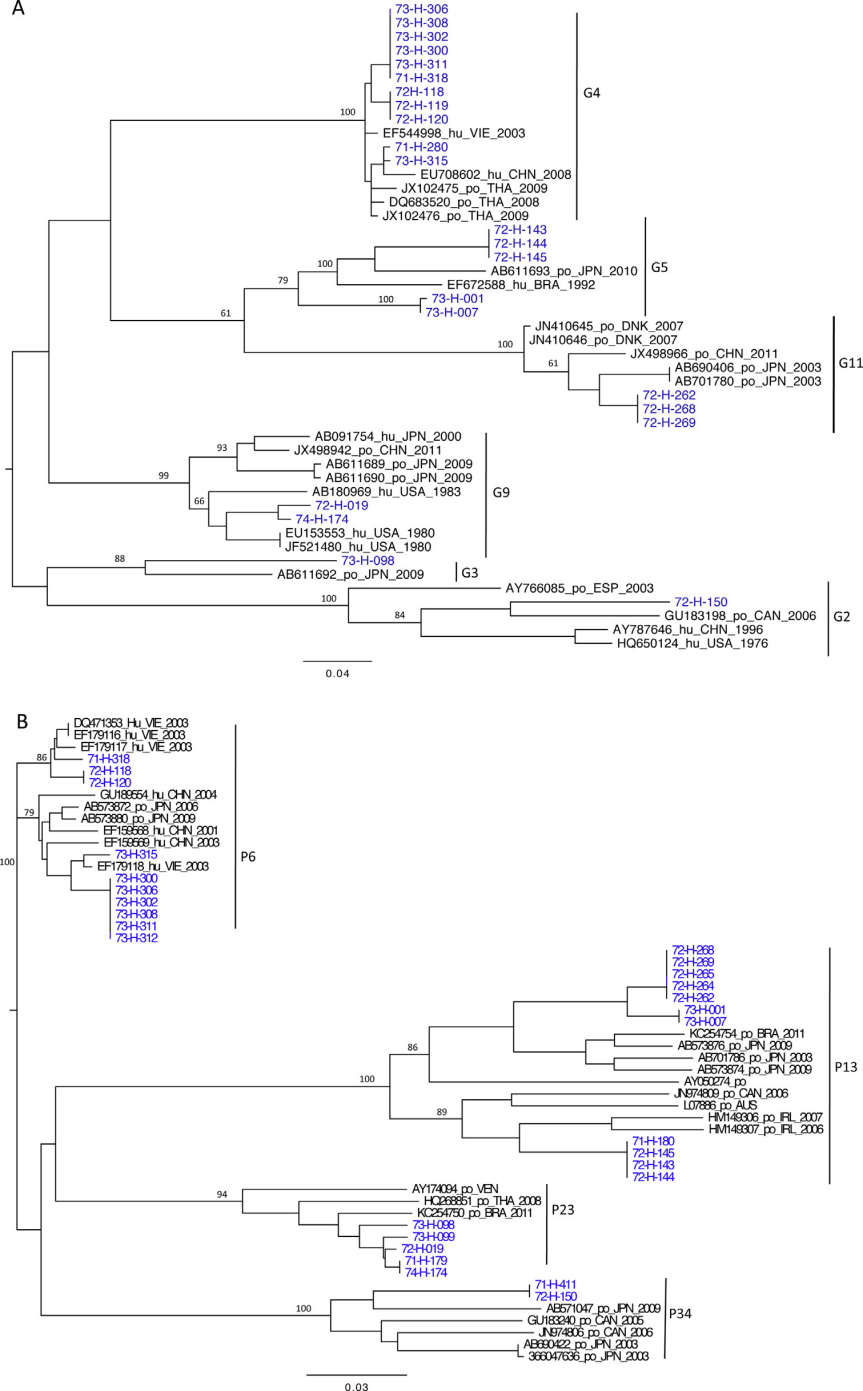
Maximum likelihood phylogeny based on partial sequences of (A) VP7 and (B) VP4 genes from Vietnamese pigs and selected reference strains. Trees were mid-point rooted; bootstrap values of 1000 trials are indicated on nodes (values > 60). Sequences generated in this study are in blue. Names of each reference sequence comprise Genbank accession number, host origin, country, and year of collection. Po, porcine; Hu, human; AUS, Australia; BRA, Brazil; CAN, Canada; CHN, China; DNK, Denmark; ESP, Spain; GBR, Great Britain; IND, India; IRE, Ireland; JPN, Japan; VEN, Venezuela.

The 24 VP7 gene sequences were analyzed using the automated online RotaC v2.0 genotyping tool to resolve the genotype assignments. A VP7 neighbor joining phylogeny was constructed using selected reference strains and a contemporary collection of closely related human and porcine sequences from Genbank (Fig. 2a). Eleven G4 strains identified from five farms were most closely related (96% nt identity) to human pediatric diarrheal cases identified in Dong Thap province in 2008–2009 (Tra My et al., 2011), to G4 strains isolates from pigs sampled Thailand (98% nt identity) (Saikruang et al., 2013), and to a G4 lineage recently identified among pediatric diarrhea cases in Wuhan, China (Wang et al., 2009). The human G4 strains previously identified in Vietnam were combined with P[8], however, 9 of the porcine G4 strains identified here were combined with P[6], and the remaining two G4 strains were P untypable. The five G5 strains were recovered from two different farms, and the most closely related G5 sequences (from Genbank) were a Brazilian 1992 human sequence, and recent Japanese and Thai porcine sequences found in combination with P[13] (Chan-it et al., 2008). Each of the porcine Vietnamese G5 strains identified here were also found in association with P[13]. The three G11 strains were identical over the aligned region and were most closely related (92% nt identity) to a lineage of porcine-human G11P[6] reassortants first identified in South America in 2009 (Banyai et al., 2009). The two sequences identified as G9 genotype clustered together with older human 1980 G9 strains from the USA (90–91% nt identity), and with more recent G9 porcine strains from mainland China and Japan (Martel-Paradis et al., 2013), for which the associated P types were P[7] and P[13].

### 3.6 Sequence and phylogenetic analysis of VP4

The 28 VP4 gene sequences were analyzed as before using RotaC v2.0. Fig. 2b shows a VP4 maximum likelihood phylogeny based on alignment of the Vietnamese porcine sequences with representative contemporary closely related sequences. The P[13]-like sequences segregated into two distinct clusters corresponding to G5 or G11 genome associations. The P[13] associated with G11 appeared most closely related to Japanese sequences from wild boars (86–88% nt identity); whereas the P[13] associated with G5 showed only moderate homology to older 1990s porcine P[13] Genbank entries (79–87% nt identity) from Ireland and Australia. None of the P[6] strains were associated with G4 and one was G untypable. Similar to the P[13]-like sequences, the P[6] strains also fell into two clusters. Both clusters of P[6] sequences were closely related (98% nt identity) to Vietnamese human isolates of porcine-like P[6] rotaviruses (Nguyen et al., 2007), and atypical human G5P[6] viruses identified in China (Li et al., 2008). The P[23] sequences had 94% identity to 2011 porcine strains from Brazil. The closest homologues in Genbank to the two Vietnamese P[34] sequences were unpublished submissions from a Japanese investigation of wild boars (FGP51, 86% nt identity), and these also showed low homologies (<71%) to porcine and bovine submissions from China. Two identical P[34] sequences were generated, one of which was associated with G2, and appeared related to a widely distributed lineage of viruses previously detected in North America and Asia.

## 4 Discussion

Our results indicate widespread endemic circulation of porcine ARoVs on smallholder farms in the Mekong Delta region of southern Vietnam, with an overall farm prevalence of 32.7%. Similar high prevalence of ARoVs has been reported from Thailand (22.3%) (Khamrin et al., 2007), Brazil (35.3%) (Rácz et al., 2000), and South Korea (38%) (Chun et al., 2010); whereas other countries have reported much lower prevalence in swine populations (e.g. 4% in Germany; 9.2% in Canada; 3% in Argentina) (Wieler et al., 2001; Morin et al., 1983; Parra et al., 2008). Notably, each of the cited studies involved detection of rotavirus in swine outbreaks, whereas the current study involved sampling of mostly healthy pigs. Indeed, there was relatively little evidence of ARoV-associated gastrointestinal disease among our surveyed farms. The high prevalence of ARoV in the absence of disease, even amongst animals with very high viral loads, suggests that positive detections from future porcine diarrheal outbreaks should be interpreted with caution. We also hypothesize that rotavirus transmission may be characterized by significant heterogeneity in virus shedding, such as the phenomenon of ‘super shedders’ as has been observed in other RNA viruses (Lau et al., 2013).

Our study showed that pigs on farms that source their water from the river had an increased ARoV infection frequency. This is consistent with previous studies where rotaviruses have been found in surface water samples at high concentration levels, confirming that river water is likely to contribute to virus transmission cycles on farms (He et al., 2012). 63% of pigs were from farms that used river water as drinking source for livestock without prior treatment. The district of Chau Thanh was also associated with increased risk. This district has over 50% of the farms and >70% of the pigs in the target population. We cannot explain the fact that density of poultry was a significant risk factor, but it is likely to be confounded by factors associated with increased density of farming where biosecurity is probably more difficult to maintain.

Studies of porcine rotaviruses in Asia suggest that the predominant ARoV G genotypes are G3, G4, G5, G9, and G11, and that they typically appear in combination with six P types (P[6], P[7], P[13], P[19], P[23], and P[26]) (Ghosh and Kobayashi, 2011). Here we detected representatives of each of these common G types, plus a G2 strain and one fragment (425 bp) with high similarity to G26. Among the P genotypes, we did not detect the purportedly common P[7], P[19], or P[26], but instead found a typical predominance of P[6], P[13], P[23], and the somewhat less common P[34]. Each of these genotype specificities have previously been found in both humans and pigs, although typically for G2, G4, G5 and G9, the human and porcine lineages of these genotypes tend to be distinct.

In Vietnam, as elsewhere in the world, the predominant strains of ARoV within human populations are G1–G4, P[4], and P[8]. A study of clinical pediatric cases in the Mekong province of Dong Thap found that G1 and G12 strains were dominant, although G2 and G3 were also detected; and among P types, P[8] was dominant, with occasional P[4] and P[6] detections (Tra My et al., 2011). With respect to livestock populations, there is no data on distribution of ARoV within pigs or any other domestic livestock of companion species, although there has been at least one report of porcine-like P[6] detections among Vietnamese pediatric diarrhea cases (Nguyen et al., 2007), and one isolation of a novel bovine-human reassortant (G10P[8]) from northern Vietnam (Matsushima et al., 2012).

Our study generated significantly lower yields of quality sequence reads and recoverable genotype data than comparable studies of porcine rotavirus in Asia (Kim et al., 2010; Chun et al., 2010). Most other surveys have sequenced from rotavirus cell-culture isolates, whereas here we performed direct sequencing of fecal suspension extracts, which may contain inhibitory substances that reduce PCR sensitivity, and/or a higher diversity of viral genomes or mixture with CRoV strains that interferes with amplification reactions. In addition, our samples were predominantly from healthy pigs, leading to lower viral loads than typically used for molecular investigations. Unfortunately, due to the difficulty in recovering sequence data from a large portion of our sample set, it remains unclear whether our data accurately represents the true prevalence of G and P genotypes in the study region. Although the majority of ARoV diversity within our collection remains uncharacterized, we conclude that the ARoV genetic assemblages are highly diverse, reflecting complex patterns of circulation.

Recent studies have indicated that certain G-P combinations are found more commonly in humans than in animal reservoirs, and vice versa (for example G2 is typically found together with P[4] in humans, whereas G2 combines almost exclusively with P[34] in swine) (Collins et al., 2010; Martella et al., 2005). These observations suggest that molecular determinants of host specificity and particular favored genome constellations may involve epistatic or viral protein interactions between the VP4 and VP7 (Heiman et al., 2008). Here were report the detection of one porcine G2P[34] strain, and an additional P[34] strain for which the G type could not be determined. Further sequence analysis of the full genome complement of these two strains may provide insight into the phenomenon of combination-specific G—P host restriction.

Studies in Europe using very substantial datasets (>36,000 genotyped human strains of RoV) suggest that approximately two percent of human clinical isolates of ARoV infection have genome constellations derived from human-animal hybrids (Iturriza-Gómara et al., 2011). Given the very high prevalence of porcine ARoV endemicity reported in this study, and the intensity of human-animal contact within the context of smallholder pig production systems in Vietnam, we hypothesize that the fraction of hybrid ARoV genomes circulating in Vietnamese human and swine populations is significantly higher than in Europe. One primary limitation of the present study was the exclusive focus on detection of ARoVs, despite the fact that additional rotavirus species (BRoV and CRoV) are known to be widespread in swine and frequently co-circulate with ARoVs. The rationale for our focus was based on the predominance of ARoV among human infections. However, in future studies we may address the broader diversity of additional rotavirus species, and explore rotavirus circulation among other in-contact domestic mammals (felines and canines).

In summary, we report that porcine ARoV infections are endemic and widespread in both healthy and diarrheic swine of the Mekong delta, and report the first sequence typing data on G and P types of porcine ARoV from Vietnam. Our study indicates that porcine ARoVs are genetically similar to those previously detected from human diarrhea cases, and thereby contributes to the growing body of evidence suggesting that interspecies transmissions and reassortment among ARoVS are relatively common. Further characterizations of whole genome sequences are required to assess the apparent cross-species traffic, and to better understand the epidemiological relevance of porcine reservoirs of viral diversity given the context of future vaccination interventions. Our results are relevant to growth of the swine industry, veterinary practitioners, and to ARoV vaccine programs in Vietnam.

## Figures and Tables

**Table 1:** Risk factor analysis for ARoV prevalence.

	Univariabel models			Multivariable model^b^		
	OR	95% CI	*p*-Value	OR	95% CI	*p*-Value
Poultry farm density^a^ (Log No. poultry farms/km^2^)	2.73	1.51–4.93	<0.001	2.45	1.38–4.36	0.020
Pig farm density^a^ (Log No. pig farms/km^2^)	1.36	1.01–1.84	0.044			
Human density^a^ (Log No. people/km^2^)	1.47	1.0–2.16	0.047			
Age of pig less than 60 days	2.89	1.63–5.12	<0.001	2.87	1.64–5.03	<0.001
Source of water						
Municipal water (Baseline = Not municipal water)	0.28	0.10–0.80	0.018			
River water (Baseline = No river water)	2.06	1.02–4.14	0.043	3.26	1.65–6.43	<0.001
Well water (Baseline = No well water)	0.84	0.38–1.83	0.654			
District of Chau Thanh	2.06	1.0–4.22	0.049	2.90	1.38–6.10	0.005
Diarrhea (Baseline = No diarrhea)	0.48	0.21–1.07	0.072			
Log (No Pigs in the farm)	1.47	0.95–2.27	0.081	1.44	0.95–2.18	0.084
Log (No. Sows in the farm + 1)	1.5	1.02–2.20	0.041			
Log(No. weaners in the farm + 1)	1.26	1.0–1.59	0.048			
Log(No. growers in the farm + 1)	0.94	0.72–1.22	0.645			
Log(No. sucklers in the farm + 1)	1.12	0.89–1.42	0.326			
Log(No. poultry in the farm + 1)	1.0	0.81–1.24	0.993			
Presence of dog (baseline = No dog)	1.36	0.70–2.65	0.37			
Presence of cat (baseline = No cat)	1.76	0.82–3.77	0.148			
Frequency of rodents sightings (baseline = No rodents seen)						
Less than once per month	0.94	0.41–2.15	0.878			
1–4 times per month	0.98	0.41–2.31	0.955			
>4 times per month	0.25	0.07–0.90	0.034			
Use of commercial feed	0.80	0.28–2.26	0.669			

a Census data used at commune level.

b Model intercept = −2.47.

**Table 2:** G and P typing data for selected Vietnamese porcine ARoV positive samples.

P type	No. of pigs with indicated genotype							
	G2	G3	G4	G5	G9	G11	G untyped	Total
6	–	–	9	–	–	–	1	10
13	–	–	–	5	–	3	3	12
23	–	1	–	–	2	–	3	6
34	1	–	–	–	–	–	1	2
Untyped	–	–	2	–	–	–	–	3
Total	1	1	11	5	2	3	8	
