# Supplementary material for: The prevalence and genetic diversity of group A rotaviruses on pig farms in the Mekong Delta region of Vietnam
Source: Vet Microbiol. 2014 Jun 4;170(3-4):258–65. doi: 10.1016/j.vetmic.2014.02.030 (PMC4003349; doi:10.1016/j.vetmic.2014.02.030)
Supplement: Supplementary file 1 [file mmc1.doc]

**Table S1.** Human population, farm statistics (per km^2^), and number of samples tested for ARoV in four districts of Dong Thap province.
